# A cross-sectional survey on the use of herbal tea among Cameroonian adults (18–65 years)

**DOI:** 10.1186/s12906-023-04040-6

**Published:** 2023-08-11

**Authors:** Abenwie Suh Nchang, Sylvia Njong, Sandra Fankem Noukimi, Lahngong Methodius Shinyuy, Sylvie Bambara, Edgar M. Kalimba, Joseph Kamga, Jacob Souopgui, Stephen Mbigha Ghogomu, Michel Frederich, Jean Lesort Louck Talom, Annie Robert

**Affiliations:** 1 Department of Epidemiology and Biostatistics (EPID, Institute de Recherche Exprimentale Et Clinique (IREC), Université Catholique de Louvain (UCLouvain), Public Health School, Brussels, Belgium; 2 Embriology and Biotechnology Laboratory, IBMM-ULB, Brussels, Belgium; 3 Laboratory of Pharmacognosy, Department of Pharmacy, Center of Interdisciplinary Research On Medicine (CIRM), University of Liege, Liège, Belgium; 4 King Faisal Hospital, Kigali, Rwanda; 5 Biotechnology Unit, University of Buea, Buea, Cameroon; 6 Aumônerie - Hôpital du Jura Bernois SA, Moutier, Suisse

**Keywords:** Herbal tea use, Experiences, Acceptability, Cameroonian adults

## Abstract

**Background:**

In respect of the WHO’s commendation to incorporate traditional medicine (TM) in health care, the Cameroon government wants to promote the use of the traditional medicine and is resolute on encouraging the treatment of patients with alternative medicine from traditional sources. This study explores the use of herbal tea by Cameroonian adults to prevent or treat diseases and the socio-demographic determinants of tea use among participants.

**Methods:**

A cross-sectional survey was conducted among 307 Cameroonian adults (18–65 years) randomly selected within 4 hospitals and 4 communities in the Centre and Southwest regions of Cameroon between 04/01–20/04/2022, using interviewer administered semi-structured questionnaires. Binary logistic regression analysis was conducted to determine the association between variables.

**Results:**

Over four-fifth (89.3%) of participants had taken herbal teas at least once within the last 2 years prior to the survey, and most participants used the teas for the prevention and treatment of Covid-19 (67.9%), malaria (59.7%) and typhoid fever (35%). Most respondents took the teas warm (75%), and the treatment dosage used by most respondents (51%) was “one glass in the morning and evening for one to two weeks”. The teas taken by 70% of users had bad or bitter taste and 52.2% of them were uncomfortable with the bad taste. However, the majority of users completed their treatment dosage (72%), 90.5% of them were willing to use teas for treatment if prescribed in health facilities in future, and 90.1% were in support that herbal teas should be prescribed in hospitals. There was no significant association (*p* ≥ 0.05) between sociodemographic characteristics of participants and herbal tea use. However, the major motivating factor for acceptability of herbal tea use was treatment effectiveness (52.7%).

**Conclusion:**

There is high prevalence of herbal tea use among adults Cameroonians in the studied settings in the Centre and Southwest regions of Cameroon, with a positive opinion and willingness to use teas if prescribed in health facilities. Authorities must ensure the effectiveness and safety of traditional medicine served in health facilities, to enhance compliance and adequate use.

## Introduction

Herbal medicines include herbs, herbal materials, herbal preparations and finished herbal products that contain, parts of plants, other plant materials or combinations thereof, as active ingredients [1]. Herbal Medicine (HM) is also known as Complementary Alternative Medicine (CAM), and it involves ways of treating and maintaining health that existed before the arrival of orthodox medicine [2]. Generally, the use of herbal medicine is very frequent in traditional medicine (TM) for the treatment of diseases. However, traditional medicine covers a wider area, where other components of nature, as well as animals and fungi can also be used for the treatment of conditions or diseases [3]. According to WHO, traditional medicine refers to the knowledge, skills and practices based on the theories, beliefs, and experiences indigenous to different cultures, used in the maintenance of health and in the prevention, diagnosis, improvement, or treatment of physical and mental illness [1]. Traditional medicine occupies a very important place in health care in Africa with a substantial prevalence of complementary and alternative (CAM) product use in the general population ranging from 4.6% in urban settlements to 94% in semi-urban settlements(Cameroon inclusive), and an estimated average of 58.2% [4, 5].

The use of herbal medicines has increased remarkably throughout the world, with many people now using these products for the treatment of many health problems in health care practice across different countries [6]. Many Cameroonians today, especially those in the rural areas and the urban poor, rely on the use of herbal medicine when they are ill; and in the rural areas, people begin by treating themselves before going to a traditional practitioner or a modern doctor [7].

The increasing widespread use of TM has prompted the WHO to promote the integration of TM and CAM into the national health care systems of some countries and to encourage the development of national policy and regulations as essential indicators of the level of integration of such medicine within a national health care system [8, 9]. Also, and according to WHO, health goals can’t be achieved without the incorporation of herbal medicines [10]. The emergence of the COVID-19 pandemic, and the dismally slow rate of vaccine access for low- and middle-income countries (LMICs) galvanized healthcare practitioners and scientists to re-visit both conventional and traditional medicines [11]. In Cameroon, four traditional medicines were approved by the government and added on the national list of essential drugs to be sold in shops to treat corona virus including; Corocur, Viro Green Force4, Elixir Covid, and Adsak Covid [12, 13]. Further to this, and in respect of the WHO commendation to integrate traditional medicine in national health care systems, the Cameroon government wants to promote the use of the traditional medicine (which has long played a role for indigenous communities throughout the country) and is resolute on encouraging the treatment of patients with alternative medicine from traditional sources. However, the use of conventional medicine is still dominant in many places in Cameroon, with less than one percent of medicines from traditional sources being officially commercialized compared to more than 90 percent of modern medicines [13]. As such, users of HM are mostly initiated through convincing information from the media, families or close contacts and friends about the efficacy of previously used herbal medicines [14], and the medicines are mostly obtainable from indigenous sources such as traditional clinics, travel agencies/express buses, individual homes and in markets [15], rather than in hospital or pharmacies. Practices of traditional medicine vary greatly from country to country and from region to region, and their acceptance and use maybe influenced by several factors, including culture, history, personal attitude, and philosophy [16], common experience, perceptions about their effectiveness, delayed medical care, and sufficient knowledge of the herbs [17–19]. Several studies have been conducted in different parts of Cameroon on the use of traditional and herbal medicines in the treatment of specific health conditions [7, 20–23], but there are no studies on the use and acceptability of herbal teas in the general adult population in Cameroon. This study therefore explores the use of herbal tea by Cameroonian adults to prevent or treat infections (diseases), and the socio-demographic determinants of tea use among participants, in order to inform authorities towards adequate decision making for optimal uptake.

## Methodology

### Study design

A cross-sectional descriptive survey was conducted to capture the use of herbal teas by respondents.

### Study sites

**Fig. 1:**
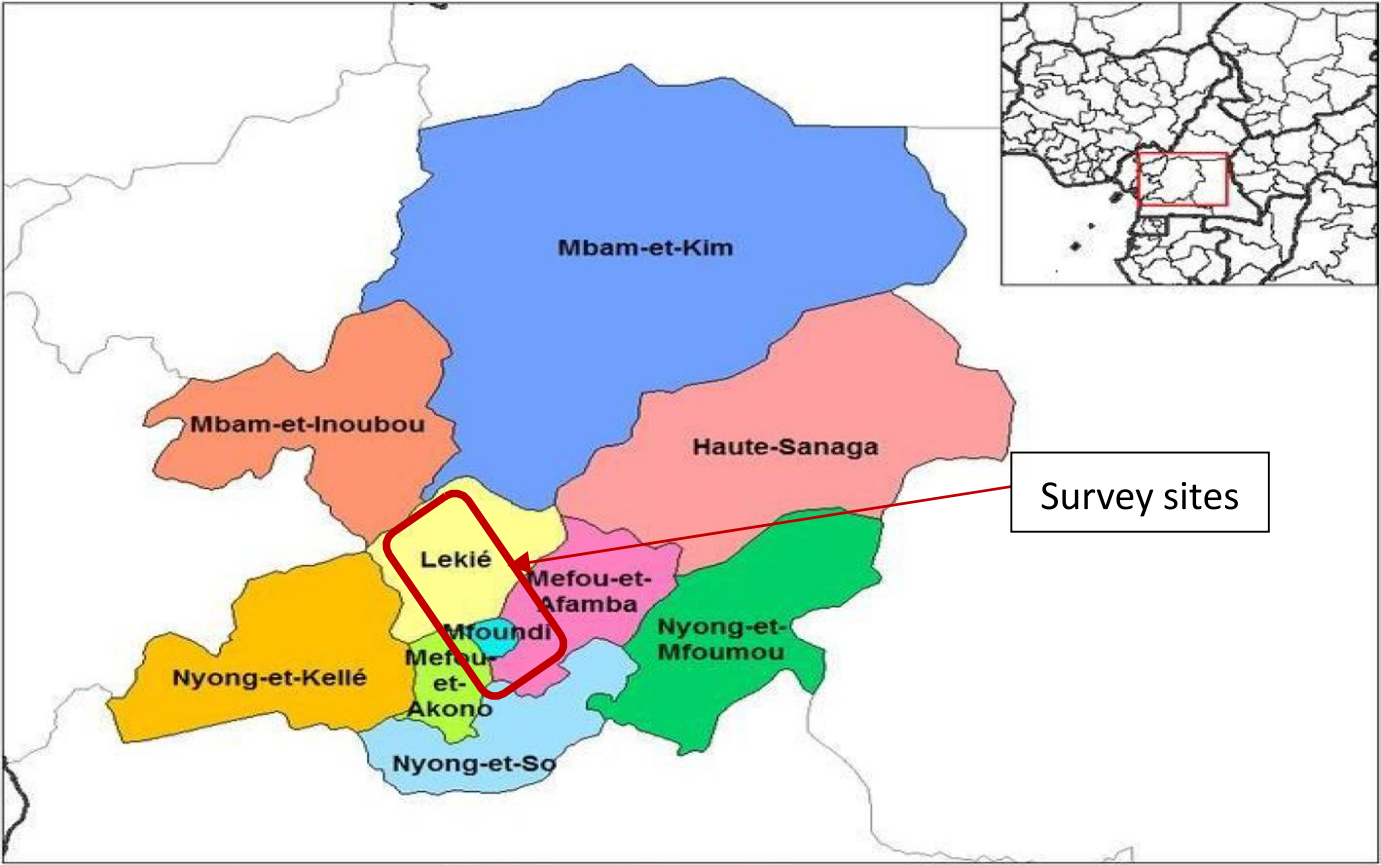
Map of the Centre region of Cameroon showing its administrative divisions and the survey sites (Source: Rarelibra 19:57, 2006)

The survey was conducted in Buea (Fako Division) in the Southwest region of Cameroon, and in Yaounde (Mfoundi and Lekie divisions) in the Centre region of Cameroon between January and April 2022, during the covid-19 pandemic. Community studies were conducted in four urban communities. These comprised; two urban communities (Muea and Mutengene) within the Buea and Tiko health districts in Buea, and two urban communities (Mbandomu and Nsimeyong) within the Biyem-Assi and Efoulan health Districts in Yaounde. Hospital studies were conducted in 2 urban hospitals (Biyem assi and Efoulan district hoapitals) and in 2 rural hospitals (Obala and Okola districts hospitals) in Yaoundé (Fig. 1).

Both community and hospital sites were chosen for data collection to capture opinions and views of respondents in both settings on the study subject and to offset the effect of environmental influence on the data acquired. Community participants are believed to naturally take herbal medicine and depend more on it for their health problems, while hospital-based participants probably depend more on modern medicines. Both groups were chosen in order to find out if the community participants would accept herbal medicine if served in the hospital or whether hospital-based respondents (who may prefer modern hospital medicine), would accept herbal medicine if served in the hospitals. Moreover, data collected in hospital settings may be subjected to desirability bias as respondents might provide a real response because of the environment. Whereas a genuine response is likely to be received in the community.

### Study area

**Fig. 2:**
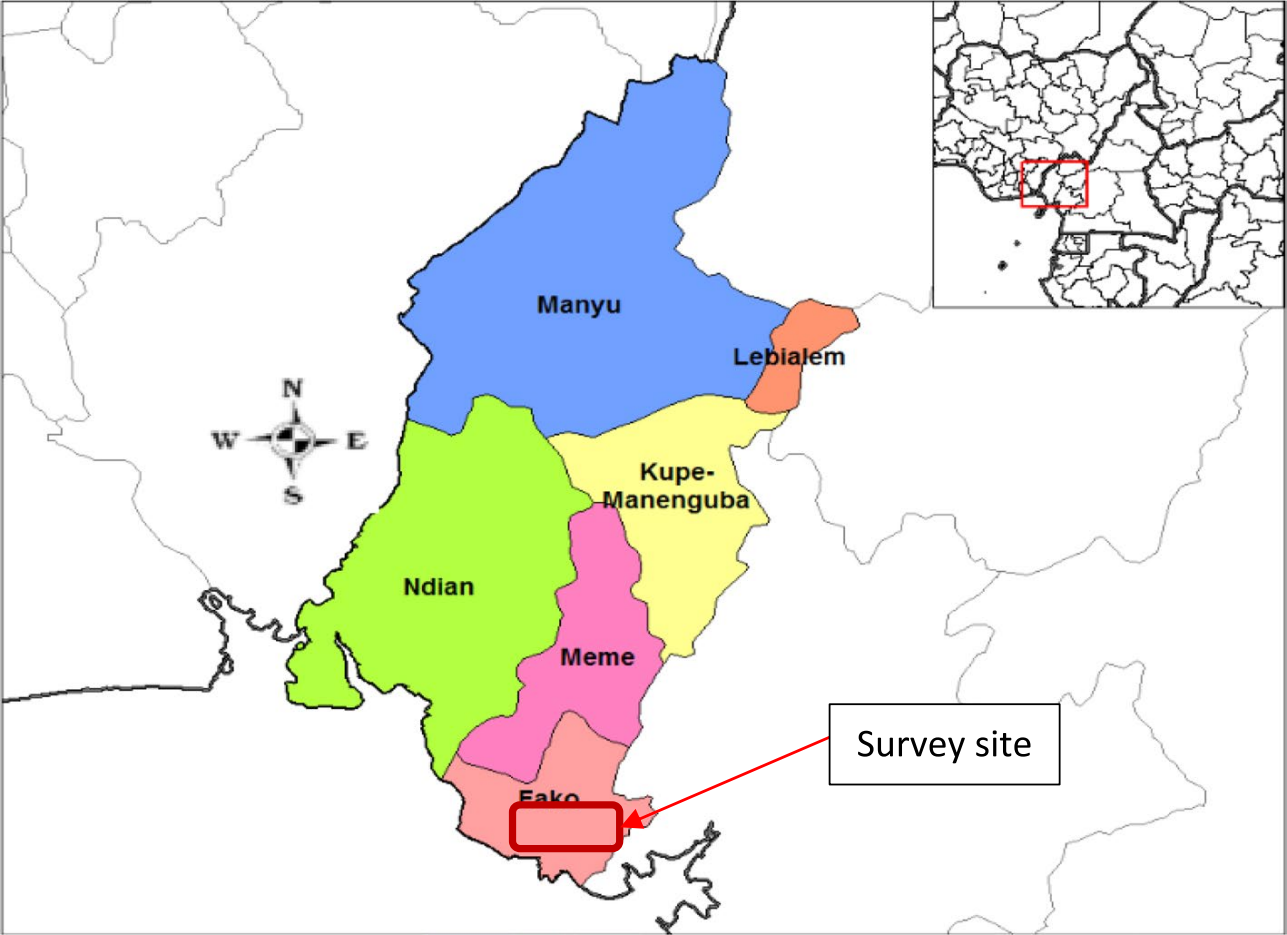
Map of Southwest region of Cameroon showing administrative divisions and the survey site (Source: Rarelibra 19:57, 2006)

Cameroon has about 90% of the African ecosystems which includes the Sahelian, Sudan, humid tropical forest, afro mountains, coastal and mountain eco-regions [24]. Fako Division in the Southwest region of Cameroon is one of six administrative Divisions in this region and is host to the Southwest regional headquarters -Buea. Other administrative Divisions in the Southwest region include Manyo, Ndian, Lebialem. Kupe-Manengubam, and Meme Divisions (Fig. 2). It lies in the coastal region between 4°28´30″N and 3°54´26″N latitudes, and 8°57´10″E and 9°30´49″ E longitudes (Fig. 2). The land area is approximately 203,071.3 hectares (ha) with a population of 534,854 people. Most inhabitants practice agriculture as the main economic activity [24]. The region has two seasons: the dry season from October to March and the wet season from April to September. People from all the three main ethnic groups in Cameroon (Bantus, Semi-Bantus, and Sudanese) are represented in this coastal region; attracted by the fertile volcanic soil for agriculture.

The Centre region is one of the 10 administrative regions of Cameroon, which lies in the southern plateau. It has a population of more than 3 million inhabitants and it is one of the most densely populated of Cameroon's regions, with a majority of its residents living in the city of Yaoundé (population 1.1 million) or along the roads and in the major towns [25]. The population density thins out away from the major thoroughfares, with few isolated settlements. While most people in the urban area are civil servants and businessmen, most people in the rural area practice agriculture. The center region of Cameroon is in-turn divided into 10 administrative divisions including Mbam-et-Kim, Mbam-et-Inoubou, Nyong-et-Kelle, Lekie, Mfoundi, Mefou-et-Akono, Nyong-et-So, Haute Sanaga, Mefou-et-Afamba, Nyong-et-Mfoumou (Fig. 2); with a total of 30 health districts (07 urban and 23 rural). It is serviced by many hospitals and clinics, particularly in Yaoundé and in the larger towns. However, traditional medicine is still common throughout, especially in the rural areas.

### Study population

The study population consisted of adult Cameroonians between 18–65 years old, resident in the study sites during the study period, who consented to participate in the survey. Hospital participants were patients and/or their care givers of Cameroonian nationality, between the ages of 18–65 years old, found in the selected hospital during the study period, who consented to participate in the survey.

### Exclusion criteria

Participants who had communication hindrances such as the deaf and mute were excluded from the study.

### Sample size calculation

Sample size was calculated using the sample size determination formula: *n* = z^2^ *p*(1 − *p*) /*d* ^2^ where n is the minimum sample size, z is the standard normal quantile corresponding to the desired level of confidence (1.96 for 95% confidence interval), d is the expected margin error (5%), and p is the WHO reported prevalence of global traditional medicine use (set at 80%) [26]. This is also close to (80.9%) found among residents of Port Harcourt, Nigeria [27]. Adapting for an expected non-respondent rate of 25%, the sample size required for the study was 307. Out of the 307 participants in this study, two-thirds (207) of them were community participants and one-third (100) were hospital participants. The number of community participants was evenly shared between the two study regions (103 in the Southwest and 104 in the Centre region. The number of hospital participants was also evenly shared in the four study hospitals (two rural and two urban).

### Sampling method: Clustering

Four health districts were randomly selected from the thirty [28] health districts in the center region of Cameroon (two among the 23 rural health districts and two among the 7 urban health districts) by lottery method in which numbers were assigned to the health districts involved, and random numbers were drawn from the pool. The district hospital of the health district selected was used for data collection. Participants within the selected health units were recruited by ***systematic sampling method***. These participants were patients or their care givers. They were recruited at the out-patient-department (OPD) and in waiting areas of the hospital. The first respondent per health unit was selected from the list of 9 initial (first nine single digits: 1–9) respondents by lottery method. Then, every—7th (digit randomly selected from 9 single digits) participant was interviewed until the required sample size for that health unit was attained. Where a participant didn’t consent to participate, the next person in line was interviewed.

At the community level, communities within the selected health districts were selected by lottery method. The starting point of questionnaire administration in each community was determined by a landmark in that community viz; The transformer at the main market in Bandoumou, HODIM church in Nsimeyong, Dr Fru’s clinic in Muea, and the police college in Mutengene). Systematic random sampling technique was then used to select households to be included in the study. The first household per community was selected from the list of 9 (first nine single digits) initial households by lottery method. Then every 4th household (digit randomly selected from 9 single digits: 1–9) was selected and an adult between 18—65 years old in the household was interviewed until the required sample size in that community was attained. In the presence of more than one adult, balloting was done to choose one adult who was interviewed.

### The study instrument

The questionnaire development was based on the study objectives, while the questionnaire design and the choice of questions was informed by the available literature from previous studies, reviews, and reports on herbal use.

The questionnaire was divided into three sections, addressing socio-demographic data, data on experiences and use of herbal tea, and data on acceptability of herbal teas if prescribed in health facilities. The questions asked were both open and close ended. The open-ended questions were used to obtain information on conditions for which the treatment was used, reasons for non-use of herbal teas (in case), Indications for use, source of herbal tea, dosage, posology, taste and ease of consumption, whether the treatment dosage was completed, opinions and acceptability of herbal teas if prescribed in a health facility, and what can increase their acceptability of herbal teas. It also allowed participants to give multiple responses to the open-ended questions.

### Data collection procedure

Data were collected using semi-structured interviewer administered questionnaires, developed based on the objectives of the study. Data were collected through face-to-face interviews, by six [6] research assistants who were trained in questionnaire administration.

### Data quality control

Intensive training was provided to research assistants (data collectors) in data collection techniques. Detailed orientation was given to the data collectors about the study before the data collection procedure started. A pre-test of the questionnaires was done on 31 (10%) of the sample population) in another community than those of the study, and the responses were checked to ensure that respondents clearly understood the objectives of the questions and provided appropriate responses to the questions, in order to validate consistency of the questions and data collection tool. Questions that were identified as not clear or irrelevant to the study were edited or omitted.

### Study variables

The outcome variables of the study were respondents’ utilization and experiences of herbal tea use, respondents’ acceptability to use herbal teas if prescribed in health facilities; while the explanatory variables were age of interviewee, gender, scholarly level, household size, profession, and socioeconomic/income status. Herbal teas in this study referred to all herbal or plant products of any kind, including the leaves, flowers, pollen, fruits, seeds, stem, or roots, weather dry or fresh; that have been prepared in the form of infusion or liquid by any means (boiling, crushing straining); and are taken orally, at any temperature (warm or cold); for the purpose of health maintenance, disease prevention or treatment.

### Ethical considerations

The study approval was obtained from the Faculty of Health Sciences-Institutional Review Board (FHS-IRB), University of Buea, Cameroon (2021/1591–01/UB/SG/IRB/FHS. Administrative authorization was also obtained from competent authorities of each study site. All methods were carried out in accordance with the Declaration of Helsinki. Each participant in the study was provided necessary information about the study including the respect of confidentiality and their right to voluntary participation. Written informed consent was obtained from all the participants. They were allowed to discontinue the interview when they needed.

### Data management and analysis

Data were checked for completeness and consistency, entered, and cleaned in Microsoft Excel, and imported into Stata 17.0 for analysis. The results were presented using frequencies with percentages in appropriate tables, and figures to display descriptive results. Binary logistic regression analyses were performed to assess the association between each explanatory variable (participant’s age, gender, scholarship level, household size, professional category, and socio-economic status) and herbal tea use. Odds ratios and their 95% confidence interval were reported and *P*-values < 0.05 were considered statistically significant. Qualitative content analysis was used to analyze the open-ended questions, and frequencies of occurrences were quantified to complement the quantitative data.

## Results

**Fig. 3:**
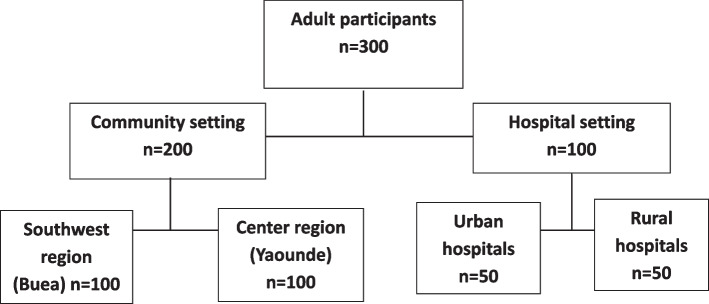
Distribution of participants across study sites

From a total of 307 participants who were identified for the study in hospital and community settings, 300 of them participated in the study and 7 of them refused to participate, yielding the response rate of 98.4%. All the 7 people who refused to participate in the study were from the community sample (3 from Buea and 4 from Yaounde). The main reason for refusal to participate in the study was a lack of interest in the study. The flow of the study participants in the different study sites is presented on Fig. 3, and distribution of the study participants according to their socio-demographic characteristics and herbal tea use, in the community and in the hospitals, is presented in Table 1.

**Table 1:** Socio-demographic characteristics of the respondents according to herbal tea use in the community and in the hospitals

	**Community respondents**		**Hospital respondents**	
**Sample Characteristics**	**N**	**%**	**N**	**%**
**Total**	**200**	**87.0**	**100**	**94.0**
***Age of respondents (years)***				
18–29	79	84.8	43	93.0
30–65	121	88.4	57	94.7
***Gender***				
Women	108	88.0	57	94.7
Men	92	85.9	43	93.0
***Scholarship level***				
Primary	48	95.8	14	100
Secondary	73	84.9	27	88.9
High school or Higher	79	83.5	59	94.9
***Size of household***	5.1 ± 2.6			
1–5 persons	129	86.1	53	92.5
> 5 persons	71	88.7	47	95.7
***Professional category***				
Student	25	68.0	16	100
Manual laborer	26	88.5	27	96.3
Officer	149	89.9	57	91.2
***Socio-economic status/Income***				
Not enough to live with	39	87.2	24	95.8
Just enough to live with	92	85.9	36	91.7
Enough and easy to live with	69	88.4	40	95.0

Size of househols min = 1, max = 15; n = Herbal tea use

### Socio-demographic characteristics of the respondents according to herbal tea use in the community and in hospitals

Table 1 shows that the use of herbal teas was generally high (> 80%) among community and hospital participants. However, the proportion of tea use was higher among hospital participants across all socio-demographic sub populations, compared to community participants, and tea use among hospital participants was higher than 91% in all but 1 category of participants (secondary education). The proportion of tea use among women was slightly higher than men for both community (88% > 85.9%) andewwwe hospital respondents (94.7% > 93%), respectively. Also, the proportion of tea use was slightly higher among participants who lived in household with more than 5 persons compared to those with household size of 5 persons or less, in both community (88.7% > 86.1%) and hospital settings (95.7% > 92.5%), respectively.

### Participants’ utilization of herbal tea

Of the 300 respondents in the survey, 268 (89.3%) had taken herbal teas within the past two years for prevention or treatment of infections, or for other purposes, while 32 (10.7%) of respondents had never taken herbal teas for any purpose. Twenty-six (26/32; 81%) of the respondents who had never taken herbal teas, were willing to take herbal teas in the future, when necessary, while 6/32 (11%) were unwilling to take teas in the future, because of lack of trust in effectiveness and hygiene of the teas (83.3) or the uncomfortable taste of teas (16.7%) (Table 2).

**Table 2:** Participants’ utilization of herbal tea

	*N* = 300
**Question asked**	**Responses**
	**n(%)**
***Have you taken herbal tea in the past 2 years for prevention, treatment or other purpose?***	
Yes	268 (89.3)
No	32 (10.7)
***If No, Can you take herbal teas to prevent or treat infections? (n*** ** = ** ***32 out of 300)***	
Yes	26 (81.2)
No	6 (19.8)
***If No, why can you not take herbal teas? (n*** ** = ** ***6)***	
Lack of trust in its Effectiveness and Hygiene	5 (83.3)
Uncomfortable taste	1 (16.7)

### Indications for use, source of herbal tea, dosage, and posology of herbal tea

Majority of the respondents who took herbal teas used them for the prevention and treatment of Covid-19 (182/268, 67.9%), followed by malaria (160, 59.7%) and typhoid fever (94, 35%). Meanwhile, 111 (41%) of participants took teas for other conditions like cough or chest infection, low blood level, to initiate labour and/or delivery, fever, common cold, worms, gastritis, knee ache, fibroids diabetes, testicular pain, mumps contraception, hemorrhoids, fertility booster, sinuses tooth ache, high blood pressure, or painful menses (Table 3). Most of the tea users received their tea prescription from someone else (65%), and 35% of teas were self-prescribed. The teas used were mainly self-prepared (37%), purchased (34%) or offered to users for free (29%). Most of the respondents took the teas warm (75%), and the treatment dosage used by most respondents (51%) was “one glass morning and evening for 1–2 weeks” (Table 3).

**Table 3:** Indications for use, source of herbal tea, dosage, and posology of herbal tea

	*N* = 268
**Question asked**	**Response**
	**n(%)**
***What condition was the herbal tea taken for? (Several options)***	
Covid 19	182 (67.9)
Malaria	160 (59.7)
Typhoid	94 (35.1)
Stomach ache	37 (13.8)
Weight loss	8 (3.0)
STIs	8 (3.0)
Others	111 (41.4)
***Did someone prescribe it to you or you prescribed for yourself?***	
Self Prescribed	95 (35.4)
Prescribed by someone else	173 (64.6)
***Did you buy it, or it was offered, or it was selfprepared?(n*** ** = ** ***268)***	
Was self prepared	99 (36.9)
Bought treatment	91 (34.0)
Was offered for free	78 (29.1)
***What quantity, how frequent, and for how long did you take it?(n*** ** = ** ***268)***	
1 glass morning and evening for 1–2 weeks	139 (51.9)
1 glass morning, afternoon and evening for 1-2 weeks	50 (18.7)
1 glass daily for 1-2 weeks	22 (8.2)
1 glass morning and evening for less than 1 week	28 (10.4)
No precise dosage	26 (9.7)
Taken until one feels better	3 (1.1)
***Did you take it warm or cold? (n*** ** = ** ***268)***	
Cold	66 (24.6)
Warm	202 (75.4)

Others: Cough/chest infections, low blood level, initiate labour and delivery, fever, common cold, worms, gastritis, knee ache, fibroids diabetes, testicular pain, mumps contraception, hemorrhoids, fertility booster, sinuses tooth ache, high blood pressure and painful menses

### Participants experiences in using herbal teas

Table 4 shows that 140/268 (52.2%) participants who used herbal teas were uncomfortable taking the teas. The teas taken by most respondents (189, 70%) had bitter taste, and 113 (42.2%) of them were uncomfortable with the bitter taste of the teas, while 60 (22.2%) of them were comfortable with the bitter taste. Even though many of the participants were uncomfortable taking the herbal tea treatment, more than two-third (193, 72.2%) of the respondents completed their treatment, mainly because of their desire to get well (181, 67.5%), and to respect the treatment dosage (12, 4.5%). On the other hand, 42 (15.7%) of respondents abandoned their treatment as soon as they felt better. Meanwhile others abandoned it because of the uncomfortable taste of the tea (24, 9.0%), because of negligence (carelessness) (4, 1.5%), because there was no precise treatment dosage (3, 1.1%), or because of ineffectiveness of the treatment (2, 0.7%). Of the 268 participants who had taken herbal tea in our survey, 244 (91%) of them had never taken artemisia tea. Meanwhile, most of the respondents who had taken artemisia tea used it for the treatment and prevention of malaria (15/24, 62.5%), or Covid-19 (5/24, 20.8%). Three-quarter 18/24 (75%) of the respondents who took *Artemisia afra* tea said it was effective for them, while 1 (4.2%) said it wasn’t effective and others 5 (20.8%) didn’t know if it was effective on them or not (Table 4*).*

**Table 4:** Participants experiences in using herbal teas

	*N* = 268
**Question asked**	**Responses**
	**n(%)**
***Were you comfortable taking the herbal tea?***	
Yes	128 (47.8)
No	140 (52.2)
***Were you comfortable with the taste?***	
It had bitter taste and I was uncomfortable taking it	113 (42.2)
It had bitter taste but I was comfortable taking it	60 (22.4)
It had no bitter taste and I was comfortable taking it	66 (24.6)
It had no bitter taste but I was uncomfortable taking it	13 (4.8)
I was uncomfortatable with its bad taste but finished it	14 (5.2)
I was uncomfortable with its bitter taste and I abandoned it	2 (0.8)
***Did you finish the treatment or you abandoned some?***	
Finished treatment	193 (72.0)
Abandoned treatment	75 (27.9)
***Why did you finish or Abandon the treatment ?***	
Finished treatment because of desire to get well	181 (67.5)
Finished treatment to respect dosage	12 (4.5)
Abandoned treatment after I felt better	42 (15.7)
Abandoned treatment because of uncomfortable taste	24 (9.0)
Abandoned treatment out of negligence	4 (1.5)
Abandoned treatment since there was no precise dose	3 (1.1)
Abandoned treatment because it was not effective	2 (0.7)
***Did you ever take Artemisia tea?***	
Yes	24 (9.0)
No	244 (91.0)
***If Yes, What condition did you take Artemisia tea for?***	
Treatment of malaria	13 (54.2)
Prevention of malaria	2 (8.3)
Prevention and treatment of Covid -19	5 (20.8)
Others	4 (16.7)
***Did the treatment work for you ?***	
Yes	18 (75.0)
I don't know	5 (20.8)
No	1 (4.2)

### Acceptability of herbal teas if prescribed in the hospital

Most respondents 266 (90.5%) were willing to use herbal teas for treatment in the future if prescribed in the hospitals, mainly because they had confidence in doctors and the hospital (137, 46.6%), because of their previous experience on the effectiveness of herbal teas (29, 9.8%), or because of their personal preferences for herbal teas (11, 3.7%). Meanwhile, other participants said they would use teas prescribed in hospital if it is the required treatment for their condition (24, 8.2%), if it is effective (57, 19.4%), if it has no side effects (4, 1.4%), or if it has been approved by health authorities (4, 1.3%). Some participants said they would not use herbal teas if prescribed in hospital because they suppose that it is not the specialty of health personnel (4, 1.4%), they do not expect teas from the hospital (12, 4.1%), or they said teas do not work for them (4, 1.4%).

The proportion of hospital respondents who were willing to use herbal teas in the future if prescribed in the hospital (95/97, 97.9%) was higher than in the community (171/197, 86.8%) All but 2 (26/28, 92.8%) of the respondents who were not willing to use herbal teas in the future were community respondents (Table 5).

**Table 5:** Participants’ acceptability of herbal teas if prescribed in the hospital

	**Whole Sample**	**Hospital**	**Community**
	*N* = 294	*N* = 97	*N* = 197
**Question asked**		**Responses**	
	n (%)	%	%
***Can you take herbal teas if prescribed in the hospital***			
Yes	266 (90.5)	97.9	86.8
***Explain your answer***			
Yes, because I have confidence in doctors/ hospital	137 (46.6)	47.4	46.2
Yes, if the treatment is effective	57 (19.4)	15.5	21.3
Yes, if it is the required treatment for me	24 (8.2)	13.4	5.6
Yes, if it has no side effects	4 (1.4)	2.1	1.0
Yes, if approved by authorities	4 (1.3)	2.1	1.0
Yes, because I know it works better, from experience	29 (9.8)	13.4	8.1
Yes, because I hate tablets and prefer herbal teas	11 (3.7)	4.1	3.6
No, I don't expect herbal teas from the hospital	12 (4.1)	2.1	5.1
No, because it is not their speciality	4 (1.4)	0.0	2.0
No, because herbal teas don't weork for me	4 (1.4)	0.0	2.0
Others	8 (2.7)	0.0	4.1

### Participants’ opinions about herbal tea prescription in hospital

The results showed that most respondents (265/294, 90.1%) where in support of herbal teas prescription in the hospitals mainly, if the teas were effective (120, 40.8%), or because they believe in doctors or the hospital (55, 18.7%); Meanwhile, some respondents simply preferred herbal treatment to tablets (17, 5.8%), and others supposed that teas are natural with less side effects (35, 11.9%), are more effective and less costly (12, 4.1%), are the same as tablets and capsules (18, 6.1%), and children could easily take them without knowing its medicine (8, 2.7%). On the other hand, some respondents were against the prescription of herbal teas in the hospital because they thought that it was not the specialty of heath personnel 25(8.5%) and there is no clear treatment dosage (4, 1.4%) (Table 6).

**Table 6:** Participants’ opinions about herbal tea prescription in hospital

	Whole Sample	Hospital	Community
**Question asked**	*N* = 294	*N* = 97	*N* = 197
		**Responses**	
	n (%)	%	%
***Will you encourage or discourage prescription of herbal teas in hospital***			
Will encourage	265 (90.1)	95.9	87.3
***Explain your reason***			
Will encourage if it is effective	120 (40.8)	27.8	47.2
Will encourage, I trust the doctors/hospital	55 (18.7)	25.8	15.2
Will encourage, its natural with less side effects	35 (11.9)	19.6	8.1
Will encourage, it's the same as tablets or capsule	18 (6.1)	11.3	3.6
Will encourage, some people prefer herbal medicine	17 (5.8)	2.1	7.6
Will encourage, its more effective and less costly	12 (4.1)	9.3	1.5
Will encourage, I like drugs in tea form and children can take without knowing	8 (2.7)	0.0	4.1
Will discourage because it is not their specialty	25 (8.5)	3.1	11.2
Will discourage because there is no clear dosage	4 (1.4)	1.0	1.5

The proportion of hospital respondents who encouraged herbal tea prescription in the hospital (93/97, 95.9%) was higher than in the community (172/197, 87.3%). All but 4 (25/29, 86.2%) of the respondents who were against the prescription of herbal teas in hospital were community respondents (Table 6).

### Factors associated with acceptability and use of herbal teas

Motivating factors for increase acceptability of herbal tea use in this study included treatment effectiveness (155, 52.7%), improved taste of teas (15, 5.1%), affordability (13, 4.4%) availability/accessibility (15, 5.1%), standardized dosage and precise prescription 13(4.4%) attractive packaging and formulation (9, 3.1%), official approval by authorities (3, 1%), and publicity /awareness campaigns (46, 15.7%). The highest motivating factor for increased acceptability of herbal tea use in both hospital and community settings was treatment effectiveness (Table 7). Binary logistic regression analysis showed that there was a high prevalence of herbal tea use in all the study sites above the WHO reported value 80%, but there was no significant association (*p* ≥ 0.05) between sociodemographic characteristics of study participants and herbal tea use in the community and hospitals study sites (Table 8).

**Table 7:** Motivating factors for increase acceptability of herbal tea use

	**Whole Sample**	**Hospital**	**Community**
	*N* = 294	*N* = 97	*N* = 197
**Question asked**		**Responses**	
	n (%)	%	%
***What can increase your acceptability to use herbal teas?***			
If effectiveness is ensured	155 (52.7)	43.3	57.4
Publicity campaigns/sensitizations/awareness creation	46 (15.7)	14.4	16.2
Others	37 (12.6)	13.4	12.2
If the taste is improved	17 (5.8)	5.2	5.1
If it is readily available	15 (5.1)	2.1	0.5
If the standard dosage and precise prescription is given	13 (4.4)	7.2	3.1
If it is affordable	13 (4.4)	8.2	2.5
Attractive packaging and formulation	9 (3.1)	5.2	2.0
If it is officially accepted	3 (1.0)	1.0	1.0

**Table 8:** Associations between socio-demographic characteristics and the use of herbal tea

Characteristics	Herbal tea use in Community settings						Herbal tea use in hospital settings				
	**Southwest region (Buea)**			**Centre region (Yaounde)**			**Urban Hospitals**		**Rural Hospitals**		
	**N %**	**OR (95%CI)**	**P**	**N %**	**OR (95%CI)**	**P**	**N %**	**P**	**N %**	**OR (95%CI)**	**P**
**Total**	100 **88.0**			100 **86.0**			50 **98.0**		50 **90.0**		
***Age of respondents (years)***			0.36			0.82		NT			> 99
18–29	46 84.8	1		33 84.9	1		23 95.6		20 90.0	1	
30–65	54 90.7	1.76 (0.52—5.97)		67 86.8	1.15 (0.35 -3.75)		27 100		30 90.0	0.96 (0.15 -6.59)	
***Gender***			0.92			0.51		NT			0.45
Women	43 88.4	1.06 (0.31—3.61)		65 87.7	1.47 (0.47—4.65)		29 96.6		28 92.9	2.05 (0.31—13.51)	
Men	57 87.7	1		35 82.9	1		21 100		22 86.4	1	
***Level of Education***			0.84			0.55		NT			0.89
Primary	24 100	-		24 91.7	2.41 (0.46—12.69)		6 100		8 100	-	
Secondary	36 83.3	0.88 (0.26—3.03)		37 86.5	1.40 (0.40—4.88)		9 88.9		18 88.9	1.14 (0.17—7.67)	
High school or higher	40	1		39 82.1	1		35 100		24 87.5	1	
***House hold size***			0.41			0.86		NT			0.16
1 -5 persons	74 86.5	1		55 85.5	1		28 100		25 84.0	1	
> 5 persons	24 91.7	1.88 (0.30—9.18)		43 86.1	1.11 (0.35—3.46)		22 95.5		24 95.8	4.57 ( 0.47—44.17)	
***Professional category***			0.14			0.18		NT			NT
Student	13 69.2	1		12 66.7	1		13 100		3 100		
Manual laborer	10 90.0	4.00 (0.37—43.13)		16 87.5	3.35 (0.44 -12.87)		19 94.7		8 100	-	
Officer	77 90.9	4.44 (1.08—18.22)		72 88.9	4.00 (0.98—16.34)		18 100		39 87.2		
***Socio-economic/Income status***	0.05					0.32		NT			0.87
Not enough to live with, but you man	20 85.0	1		19 89.5	1		13 100		3 100	1	
Just enough to live with	43 81.4	0.77 (0.18—3.28)		49 89.8	2.38 (0.44–12.87)		19 94.7		8 100	0.54 (0.04—6.58)	
Enough and easy to live with	37 97.3	6.35 (0.61—65.66)		32 78.1	2.46 (0.71 -8.58)		18 100		39 87.2	0.57 (0.05–7.00)	

## Discussion

This cross-sectional survey was aimed at exploring the use of herbal teas among adult Cameroonians (18-65 years old) in study settings in the Centre and Southwest regions of Cameroon, on the use of herbal teas, and their acceptability to use these teas if prescribed in the hospital. Out of a targeted 307 randomly selected participants from hospitals and communities in two studied regions of Cameroon (Centre and South-west), the findings provided herein are reflections of 300 respondents:100/100 (100%) hospital respondents and 200/207 (96.6%) community respondents.

### Participants utilization of herbal teas

In this study over four-fifths of participants reported to have taken herbal teas at least once within the last 2 years prior to the survey, thus indicating a high consumption rate. In addition, there is a potential for increasing prevalence of herbal teas use in the future, as many of the respondents who had never taken herbal teas were willing to take teas in the future if necessary. The high prevalence of herbal tea use observed in this study is similar to 76.7% Baluchi, Nigeria [29] 80.9% in Port Harcourt [27] and 85.1% in Plateau State, Nigeria [16]. However, this prevalence was higher than 58.2% obtained in Sub Saharan Africa [4], 60.0% TCAM reported in Zimbabwe [30], 55.5% in Malaysia [28] and reports from previous studies in Cameroon [15, 21, 31, 32]. The higher prevalence of herbal medicine use observed in our study compared to other studies in Cameroon may be because it was conducted in the general population, while the other studies were conducted in specific study populations (for specific health conditions). For example, the study conducted by Leke et al. in 2022 in pregnant women reported the prevalence of 76% [22]**.** Pregnant women are expected to use less medication and less herbal teas than the general population. In another study conducted by Ayima et al. in 2021, assessment of herbal medicine use was specific for COVID-19 treatment, with prevalence of 68.5% [21].

The high prevalence of tea use in the present study could also be due to an increased consumption of herbal tea as a precautionary measure during the emergence of the COVID -19 pandemics within the past three years which threatened the lives of many individuals and caused them to increase their health seeking behavior, including the use of traditional medicines. This is confirmed by findings of the current study where, the purpose for which most respondents used herbal teas was prevention and treatment of Covid-19, followed by malaria and typhoid fever. Unlike our findings, most of the respondents in a study in Nigeria [16] used traditional medicine (TM) mainly in the treatment of malaria and fever. Generally, people used herbal medicines to restore, promote and maintain health, as well as treat and prevent illness [28]. In line with existing data [33], the respondents in this study reported to use plants and parts of plants for different ethno-medical purposes such as antiseptic, laxatives, purgative, anticonvulsant, expectorants, anthelmintic, and sedatives in the treatment of malaria, high blood pressure, diabetes, rheumatism, diarrhea, infertility, jaundice, dysentery, gonorrhea, fever, pain, respiratory problem, or poultice, among others.

The proportion of herbal tea use in this study was higher among hospital respondents than in community respondents, across all the socio-demographic sub populations. This finding is different from our expectation that community participants would naturally take herbal medicines and depend more on them for their health problems, while hospital-based participants probably prefer and depend more on modern medicines. The high tea use among hospital respondents might be a reflection of a general health seeking attitude among respondents in hospital settings. It is equally suggestive of a probable use of herbal teas alongside conventional medications by the hospital respondents, which could result in a possible drug herb interaction that could dangerously amplify or reduce the potency of co-administered conventional medicines. Thus, there is a need for Health care providers in these settings to always inquire about the use of herbal medicines when providing health care. A study in Bauchi, Nigeria revealed that over one-third of participants combined herbs with orthodox medications and over four-fifth of them did not inform their doctors that they used herbs [29]. In line with reports from previous surveys that females are more prone to use Complementary” “Alternative” medicine (CAM) than males [27, 34–37], the proportion of herbal tea use among females in this study was slightly higher than in males, for both community and hospital respondents in the current study. Women consider traditional medicine to be safe for their health, especially in consideration of their reproductive health, including pre-natal and post-natal health, pregnancy outcomes and breastfeeding [38, 39]. In the current study women used herbal medicines to suppress menstrual pain, to boost fertility, to improve lactation, to improve course of pregnancy, or to facilitate labour.

A higher prevalence of herbal tea use was observed in respondents from homes with larger household size (> 5persons in households). This might be due to financial difficulty to provide modern healthcare to all members in large households, compared to herbal medicine which could be home prepared, received for free, or locally available at a far cheaper cost [15]. With the persistent financial hardship affecting many households in Cameroon, it is likely that many will preferably not go for laboratory diagnosis of their illness but will buy locally available and cheap medications regardless of the source and quality, if they are told it treats what they are assumedly suffering from, thus revamping their hope.

In this study, about two-thirds of the tea users received their tea prescription from someone else and one-third of the teas taken were self-prescribed. These findings were in line with findings among residents of Port Harcourt in Nigeria [27], where respondents obtained information about herbal medicine mainly from home, when family members, friends and associates shared information, knowledge, and perceptions about herbal medicine among themselves, followed by mass media and other sources. Thus, the family has a major influence in creating awareness on herbal medicines. A danger is that the family and friends may not possess the correct knowledge and information to advise relatives on the use of herbal medicine. There is a need to enlighten the population with adequate information related to herbal medicines and the dangers of their practice.

Findings that the teas taken by respondents in the current study were mainly self-prepared, purchased or offered to users for free, corroborate with data from a similar study conducted in Cameroon [15], where more than half of the study population bought their traditional medicines from inter-regional Express Buses, while others obtained them directly from traditional clinics, homes, and markets. In the same way, herbal teas used by most participants in a study in Nigeria [16] were locally sourced from farms, gardens or bought from the markets.

The observed high response in acceptance and purchase of prescriptions from local sources in the present study could somewhat be due to increasing adverts on local radios, televisions, and express buses, with the notion that traditional medicine cures all illness, is safe for use, with no adverse effects, or is cost effective. This high trust and easiness to accept and use herbal teas prescribed by other persons is a promising attitude towards acceptance of future herbal treatment in an integrated healthcare system. However, this may be dangerous to the health of the population in situations where safety and effectives of the proposed treatments are not verified. There is a need to educate the population to be precautious in accepting unverified treatment from unknown sources. There is also a need to control advertisements and misleading health related information shared by untrained drug vendors on various communication channels within the national territory.

The finding that most of the respondents in this study took the teas warm and the treatment dosage used by most respondents was “one glass morning and evening for one-to-two weeks” concord with results from Jos South Local Government Area of Plateau State, Nigeria [16], where the herbal medicine used by respondents had specified dosage indications. The above mentioned frequently used posology is apparently convenient for both students, officers, or manual laborers; and it is worth considering when prescribing herbal teas in future. Most people usually leave home in the morning for various activities and only return home in the evening, making it more convenient to take the teas when they are at home, especially as they need to take it warm.

### Participants experiences in using Herbal teas

This study revealed that the teas taken by two-thirds of respondents had bitter taste and some of the participants were uncomfortable with the bitter taste of the teas, while others weren’t bothered by bitter taste. Even though many participants were uncomfortable taking the herbal tea treatment, more than two-thirds of the respondents completed their treatment dosage because of the desire to get well and to respect the prescribed dosage. These results reveal an encouraging compliant practice by participants, and a determined wellness mentality towards treatment, irrespective of the taste or discomfort of the treatment. However, the results that more than one-tenths of respondents abandoned their herbal treatment as soon as they felt better indicate a need for increased health education to the population on the importance of taking the prescribed dosage or adhering to treatment. Also, the reasons for non-adherence revealed by respondents should be considered to improve treatment options and approaches in the future.

More than nine-tenths of the participants who had taken herbal tea in the current study had never taken artemisia tea. Those who had taken artemisia tea used it for the treatment and prevention of malaria and Covid-19. In recent reports, the Madagascar Institute of Applied Research reported an important use of Artemisia *annua* (sweet wormwood) in COVID-19 [40]. However, many of the respondents who had taken *Artemisia afra* tea said it was effective for them*.* The very low use of artemisia tea reported in this study may not be real, as most people who denied ever taking the tea, were not also familiar with the name “*Artemisia afra*”, and might have taken it without knowing its name, given that they took some teas without knowing the names.

### Acceptability of herbal teas if prescribed in the hospital

The results of this survey that more than nine-tenths of respondents were willing to use herbal teas for treatment in the future if prescribed in the hospitals, because of high confidence in doctors and the hospital, previous experience on the effectiveness of herbal teas, and preferences for herbal teas, depicts a high level of confidence in health facilities, and a positive opinion about herbal teas by our study population. This is a promising indicator of high acceptability and compliance with herbal teas if prescribed in hospital especially if the safety and effectiveness of the treatment is ascertained. However, the data also indicates a strong influence of health care providers on the potential acceptability of the teas, based on their knowledge, conviction, and willingness to prescribe the teas for patient treatment. There is therefore the need for health authorities to verify and ensure that detailed pharmacotherapeutic data on all traditional medicines of interest is made available to health care providers before their integration into the health care system. This above finding agrees with other works in Cameroon [15] and Nigeria [27], where most people perceive traditional medicines as efficacious, cost-effective and a viable alternative to orthodox medicine. This observation agrees with the general belief among most people that herbal medicines are effective, affordable, and safe [41]. Herbal drugs are less costly probably because of the less cost involved in their production. The cost-effectiveness and alleged safety of herbal medicines probably account for the high prevalence of usage of herbal medicines [41]. The misconception by respondents in this survey that herbal tea prescription is not the specialty of health personnel and should not be served in hospitals, indicates the need for more communication and related awareness education during patient care. Meanwhile, the circumstances for consideration of herbal tea treatments specified by respondents should be considered when reviewing strategies to improve acceptability to use herbal teas in the future.

### Factors associated with acceptability and use of herbal teas

Findings of this study showed no significant association (*p* ≥ 0.05), between sociodemographic characteristics of the study participants and herbal tea use in both community and in the hospital settings. This absence of a significant association must be linked to the high prevalence of tea use noted in all the socio demographic sub-populations in this study. As noted earlier, the emergence of the COVID -19 pandemics within the past three years which threatened the lives of many individuals and caused them to increase their health seeking behavior, including the use of traditional medicines might have been responsible for the evenly high use of teas among participants in all the study sub populations. Contrary to our findings, results of a similar study in Cameroon [21] found that age > 41 years, being a farmer, having an income level between 185–370 USD, residing in a rural setting, and being knowledgeable on TCAM, were predictors for TCAM use. Meanwhile being married or having low-income status were found to be positively associated with herbal medicine use among Malay women In Malaysia [28]. Findings of this study that treatment effectiveness was the major motivating factor for acceptability and use of herbal tea is satisfactory, as it is an expected wellness mindset during treatment. This concords with findings in Accra, Ghana [14], where the effectiveness and persona l preference for herbal medicines, were reported as the motivating factors for herbal tea use.

### Strengths and limitations

Contrary to many studies conducted on the use of TM in Cameroon, which are focused on a specific health condition, this survey was the first to explore the use of herbal teas in a general adult population, involving both community and hospital respondents, and with a high response rate. Unfortunately, we could not capture data on the specific types and names of teas consumed/taken by the participants within the context of the current study since the study was not focused on a specific health condition and it was broad to capture such data on all the conditions for which each participant used herbal teas**.** The traditional practices and actual traditional medicines of the indigenous people were not equally captured in this study. Therefore, further research is needed in these areas, which are of considerable interest.

This study employed a cross-sectional design which does not provide information on participants’ behaviour over time, establish cause-and-effect relationships, nor guarantee the study to be representative. There was also the potential for recall bias as the data collected was self-reported. There was probable social desirability bias given that herbal tea use is likely considered as a socially undesirable attitude and behavior, especially for respondents interviewed at health facilities.

Notwithstanding, findings of the study are of great significance as they uncover the views and experiences of participants on herbal tea use and inform authorities on some aspects to consider for improved uptake and adherence, when considering strategies to successfully integrate traditional medicine in the national health care system.

## Conclusion

In conclusion, this study revealed a high prevalence of herbal tea use among adults Cameroonians in the survey settings in the Centre and Southwest regions of Cameroon, with a positive opinion and willingness to use herbal teas prescribed in hospitals. However, there is a need for health authorities to ensure the effectiveness of traditional treatment served in the hospital, to watch out for counterfeit, verify the efficacy and safety of these herbal teas before they are allowed to be used in the hospital and community, and to ensure provision complete and precise information to patients, in order to enhance compliance and adequate use. There is also a need to verify and ensure availability of pharmacotherapeutic information on the teas to health care workers, before their incorporation in the health care system.

## Data Availability

The datasets used and/or analysed during the current study are available from the corresponding author on reasonable request.
